# How can “small childcare” support “great happiness”? A study on inclusive childcare services for infants and toddlers aged 0–3 in Guangxi through the lens of the capability approach

**DOI:** 10.3389/fpubh.2025.1664735

**Published:** 2025-10-14

**Authors:** Zhoupeng Chen, Junzhu Liu, Chunhui Li, Qing Kang, Yi Tang

**Affiliations:** ^1^School of Politics and Public Administration of Guangxi Minzu University, Nanning, Guangxi, China; ^2^School of Public Administration of Guangxi University, Nanning, Guangxi, China

**Keywords:** children aged 0–3, inclusive childcare services, capability, balanced population development, policy optimization

## Abstract

**Background:**

Under the “three-child” policy, the importance of childcare for infants and toddlers aged 0–3 has been emphasized. However, the implementation of universal childcare policies still faces problems such as supply–demand mismatches, uneven resource distribution, and insufficient supervision, which constrain families’ childcare capabilities.

**Research objective:**

Drawing on Amartya Sen’s capability approach and taking Guangxi as the research area, this study analyses the dilemmas and challenges in the implementation of universal childcare policies, explores their impact on families’ economic, social, and informational capabilities, and proposes corresponding optimisation paths.

**Research methods:**

The current implementation of universal childcare in Guangxi was systematically examined through a mixed-methods approach, combining questionnaire surveys with in-depth interviews.

**Findings:**

The results show that the policy has improved families’ childcare capabilities to some extent. However, its effectiveness has not been fully realized due to irrational resource allocation, limited service capacity of childcare institutions, insufficient family capabilities, and imperfect mechanisms of collaborative governance.

**Conclusion:**

Based on these findings, this paper proposes high-quality development paths including more precise financial subsidies, diversification of childcare supply, and strengthening of collaborative governance systems. These measures aim to enhance families’ childcare freedom and promote the goal of ensuring that every child has access to care.

## 1 Introduction

Population issues have long represented fundamental, structural, and strategic challenges on a global scale. Globally, demographic transition and the alignment of public service provision have become major family policy concerns for countries and regions worldwide (1–3). In China, with the full implementation of the ‘three-child’ policy and the deep adjustment of the population development strategy, 0–3 childcare services have emerged as a key measure to address demographic challenges, encourage fertility intentions, and promote the long-term balanced development of the population (4–6). From the demand side, data from the National Health Commission indicate that China currently has approximately 30 million children under the age of three, with more than 30% of families expressing demand for childcare services. Notably, rising urbanization and shrinking household size have undermined the sustainability of the traditional “intergenerational care” model, leading to a growing demand for modern, community-based childcare services (7). However, from the supply side, notable gaps remain in China’s childcare service system. By the end of 2023, China had 3.38 childcare places per 1,000 population, amounting to a total of 4.77 million places, with an overall enrolment rate of only 7.86 per cent. Both historically and internationally, China’s current enrolment rate remains relatively low. The gap between the limited supply of high-quality childcare resources and families’ rigid demand has become increasingly pronounced, constituting a major bottleneck that constrains the effectiveness of the birth policy.

As the region with the largest ethnic minority population in China, the only one connected to ASEAN by both sea and land, and the sole autonomous region that is coastal, riverine, and borderland, Guangxi serves as a representative sample in demographic terms (8). From the perspective of policy practice, enhancing the fertility support policy framework and unlocking fertility potential have become core initiatives in Guangxi’s strategy of safeguarding, consolidating, and revitalizing border areas in the new era. In recent years, driven by accelerated urbanization and the rural revitalization strategy, urban and rural families in Guangxi have exhibited increasingly diverse and differentiated needs for inclusive childcare services. Despite a large population of children under age three, Guangxi’s overall childcare enrollment rate remains low, with limited coverage of inclusive services. The supply–demand gap is significant, making it difficult to meet families’ basic expectations for childcare that is affordable, dependable, and reassuring. A significant shortfall in childcare slot availability highlights the misalignment between service provision and the enhancement of families’ childcare capabilities. Therefore, delivering more flexible and locally tailored childcare services has become a critical issue that Guangxi must urgently address.

Based on this, this study takes Inclusive childcare services for infants and toddlers aged 0–3 in Guangxi as its research focus and sets the following objectives. First, through a questionnaire survey of parents of children under 3 years old in Guangxi, as well as in-depth interviews with parents, heads of Inclusive and private childcare institutions, staff from the Health Bureau’s childcare department, and representatives of social organizations, we systematically examine the current development of Inclusive childcare services in Guangxi and identify key problems and bottlenecks. Second, we localize and apply the capability approach to the field of childcare services, moving beyond the traditional supply–demand binary framework to uncover the mechanisms shaping families’ childcare decisions and to assess the actual effects and challenges of implementing inclusive childcare policies. Third, based on the above analysis, we propose flexible and tailored pathways for improving childcare services that reflect Guangxi’s local characteristics and enhance families’ capabilities, thereby providing both theoretical foundations and practical approaches for strengthening the fertility support policy system, improving service quality, and promoting the long-term balanced development of the population. By achieving these goals, this study aims to make “small childcare” a key pillar for improving people’s well-being and advancing social equity. This will not only help to strengthen Guangxi’s childcare service system, reduce the childcare burden on families, expand access to inclusive and high-quality services, and enhance people’s sense of well-being and fulfillment, but also provide transferable experiences and models for other regions.

The main contributions of this study are threefold. Theoretically, it introduces the capability approach into the study of childcare policy, thereby expanding the analytical lens for policy evaluation. From a policy perspective, it identifies the practical challenges facing inclusive childcare in Guangxi and proposes capability-based strategic recommendations, offering insights for government policy formulation. In terms of practical value, it offers replicable insights for the promotion of inclusive childcare in other less developed regions.

## 2 Literature review

### 2.1 Research on the supply and demand of inclusive childcare services and the factors that influence them

Foreign research on childcare services began earlier and exhibits distinctive multidimensional characteristics, with a core focus on systematic exploration from multiple perspectives, including policy, service models, quality assessment, and social effects (9–11), emphasizing the long-term effects of childcare services on children’s cognitive, emotional, and social development through empirical analyses (12–14). Regarding research on influencing factors, Blumenberg et al. (15) assessed childcare accessibility using the two-step floating catchment area (2SFCA) approach, which accounts for geographic, socio-demographic, and employment factors, providing a more precise evaluation of accessibility disparities across different community types. Yerkes and Javornik (16) conceptualized and evaluated childcare policy design based on five key dimensions—accessibility, availability, affordability, quality, and flexibility—from a national comparative perspective, analyzing childcare systems in six countries and drawing on Sen’s capability approach. Natalia et al. (17) employed a natural experiment framework to examine the impact of childcare policies on the quality and availability of services in Spain, where female labor force participation was low and childcare infrastructure was inadequate. Their findings highlight the effects of providing full-day public childcare for three-year-olds in such a context. This study provides key evidence for assessing the impact of inclusive childcare policies in economically disadvantaged environments, emphasizing the necessity of designing policies that target specific groups (e.g., older mothers or those with larger families). Moerk et al. (18) leveraged exogenous changes in user fees brought about by childcare reforms in Sweden and employed a natural experiment to precisely identify the causal effect of childcare costs on fertility. By analyzing differences in cost schedules across cities and exogenous changes in family characteristics, their study provides micro-level evidence for optimizing inclusive childcare policies in high-welfare countries. It also emphasizes the need for policy design to balance cost reductions with economic incentives for families. In addition, relevant scholars have explored how to build a sustainable service system from the perspective of the perfection of policies and regulations and the professional management of childcare institutions, further deepening the research on the linkage mechanism between childcare services and population policies, which not only reflect the differences in cultural traditions and social structures among countries, but also provide diversified theoretical frameworks and practical experiences for the construction of China’s childcare system.

Under the context of the official launch of Chinese-style modernization and the years-long implementation of the comprehensive “two-child” policy, the influencing factors of “third-child” fertility intentions and childcare choices have inevitably exhibited new characteristics. These factors are closely intertwined with policy orientation, reflecting not only the urgent demands of demographic transition but also the deep-rooted contradictions in the distribution of family and social resources (19–21). With demographic changes, rising levels of economic development, and changes in family childcare concepts, more and more families have begun to reevaluate the relationship between childbearing decisions and childcare arrangements. Yan Jiyao et al. (22) used ArcGIS spatial analysis to explore the spatial differentiation characteristics of childcare institutions in China, and used geographically weighted regression to reveal the influencing factors of spatial differentiation, and found that childcare institutions in China generally show an agglomeration-type distribution pattern with more in the east than in the west, and a significant spatial distribution characteristic of “large agglomeration and small dispersion.” The number of childcare centers is positively correlated with the level of the city. Hu Xiwu et al. (23) conducted an empirical analysis based on 1,143 questionnaires from eight cities and towns in Qinghai Province, using the SHAP algorithm and logistic regression model to explore the influencing factors and mechanisms of women of childbearing age’s willingness to give birth to “three children” in Qinghai Province. The results show that ethnicity, education level and sex of the child are the main factors influencing women of childbearing age’s willingness to have three children in Qinghai Province. Individuals must consider the costs of childbearing, which come from the expenses of childbirth, parenting and education. Studies have shown that an individual’s economic status significantly affects the willingness to have two or more children (24–26). Economic factors aside, widely discussed social issues are also important causes of the low fertility phenomenon (27). In general, with the development of the economy and society, the main factors affecting fertility decision-making have changed from the country’s overall population policy to endogenous factors such as individual income, education level, family generation, and low-fertility culture (28, 29). Fertility decline is the result of the combined effect of the macro level of social development and the micro level of increased individual burden.

### 2.2 On the theory of capability approach and its application to childcare

Sen first introduced the term ‘capability approach’ in 1980 and further refined its definition in 2002 as ‘the set of functionings an individual possesses’ and ‘the genuine freedom to pursue a life he or she reasonably values’ (30). Arguably, the capability approach involves an individual’s pursuit of freedom, which entails transforming one’s potential capabilities into actual functioning (31). The concept of the capability approach consists of two core components: functionings and capabilities. First, functionings refer to the actual achievements or states of being that a person attains. Secondly,“capacity,” or “viability,” refers to the overall quality of a person’s performance in a task (32). It is emphasized that welfare is not created by the goods themselves, but by the opportunities and activities they bring (33). A person’s capability approach is the freedom to make choices in different areas of life (30, 34, 35). This approach takes functionality and capability as its core concepts and emphasizes that the most important goal of development is to “expand people’s capabilities,” not just economic growth. Building on this concept, Sen proposes five instrumental freedoms essential for development: political freedoms, economic facilities, social opportunities, transparency guarantees, and protective security. These five instrumental freedoms complement one another, expanding individuals’ capabilities and substantive freedoms. Public policy plays a crucial role in fostering these interconnected freedoms (36). Heckman (37) emphasizes the impact of early education on children’s cognitive, noncognitive skills (e.g., social competence, emotional regulation, etc.), especially for low-income families’ children, and offers targeted policy recommendations aimed at promoting social equity and economic development by upgrading the skills of children, especially those living in poverty. The viability analysis approach is widely used to assess the welfare status of individuals or groups in terms of inequality, poverty, health, etc. (38), and is an alternative tool for analyzing social cost-effectiveness and a normative analytical framework for evaluating the effects of policies (39–41).

### 2.3 Analytical framework: based on the capability approach

Historically, economic growth has been treated as the sole metric of social progress. However, individual well-being depends not only on access to material resources but, more importantly, on the freedom to transform those resources into a life one has reason to value. Against this backdrop, Amartya Sen introduced the concept of capability in 1980. The capability approach underscores individuals’ real freedoms and abilities to pursue lives they consider meaningful. It moves beyond the mere distribution of material goods to examine how individuals can actually use resources to achieve their goals (30). In the context of current childcare policy, many families experience a clear form of capability deprivation. This is reflected in various dimensions: limited financial capability due to economic constraints; constrained decision-making power due to information asymmetry; and weakened trust capability due to concerns about service quality. These overlapping deficiencies serve as root causes of structural imbalances in the childcare market and persistently low enrollment rates. Traditional childcare services have primarily emphasized basic caregiving and safety. However, guided by the capability approach, modern childcare increasingly focuses on supporting children’s multidimensional development—cognitive, emotional, social, and physical—thereby expanding their future life opportunities and freedom of choice.

Advancing an inclusive childcare system constitutes a complex collective action challenge involving multiple stakeholders, including governments, childcare providers, and families. This paper adopts the capability approach as a theoretical lens to construct a three-dimensional analytical framework: (1) the resource dimension, which concerns reducing childcare-related costs and ensuring basic accessibility; (2) the freedom dimension, which addresses the expansion of substantive freedoms—especially for women—in career development and time autonomy; and (3) the development dimension, which aims to realize individual potential and promote social equity. Through this framework, the study explores how inclusive childcare contributes to the enhancement of collective capabilities and the promotion of long-term social well-being.

By introducing Amartya Sen’s capability approach, this study introduces a ‘developmental childcare’ paradigm that surpasses the limitations of traditional childcare services focused solely on basic care. The study advocates for childcare services should shift from survival protection to developmental empowerment. By providing rich learning opportunities and emotional support, childcare services can help children develop their potential abilities. By enhancing the capability approach of childcare services, it can ensure that more families have access to high-quality and affordable childcare services, especially for the relatively less well-off families. Inclusive childcare focuses not only on the universality and accessibility of services, but also on the professionalism and safety of service content, so that childcare resources can truly benefit all children, contributing to the enhancement of social equity and family well-being, and strengthening their freedom of choice in the future. This combination helps to promote the overall development of society and the formation of a more harmonious and sustainable childcare ecology, which can truly enhance the long-term well-being of children and families, and this innovative understanding of the concept of well-being provides an operational research framework for promoting empirical and normative research on inclusive childcare systems.

To summarize, existing research has extensively discussed the supply and demand of inclusive childcare services, influencing factors and other related issues, but there are still the following shortcomings: on the one hand, the relevant literature focuses on the current situation of the supply of childcare services, problems and the evaluation of the effects of the birth support policy, but there is a lack of in-depth exploration of the model of inclusive childcare. In particular, there is a lack of research that systematically deconstructs how inclusive childcare services can realize the development of individual capabilities and the enhancement of family well-being from the perspective of the capability approach. Against this backdrop, the intrinsic linkage mechanism between “small childcare” as a specific social service initiative and the macro social goal of “big happiness” has not been fully explored. Further exploration of this issue will not only help deepen theoretical understanding, but also provide more targeted support for the formulation and implementation of relevant policies. Moreover, research on inclusive childcare services in underdeveloped regions of western China remains limited. As a typical multi-ethnic and underdeveloped province, Guangxi explores the breakthrough of resource constraints through policy innovation, and it is of practical urgency and significance to conduct empirical research on the age-appropriate population of childcare services and resource demand for the construction of a childcare service system in ethnic areas, whose experiences can provide valuable insights for similar regions, facilitate the expansion and quality improvement of inclusive childcare services, and ultimately contribute to balanced regional development and enhanced social well-being.

## 3 Research design

### 3.1 Research methods

Guided by the capability approach, this study focuses on Inclusive childcare services for children aged 0–3 in Guangxi. A mixed-methods design was employed, combining questionnaire surveys and in-depth interviews. The questionnaires were distributed through multiple channels, including universities, kindergartens, nursery schools, trade unions of public institutions and state-owned enterprises, neighborhood committees, and residential property management offices. The survey enabled large-scale data collection, covering residents of different ages, educational backgrounds, occupations, and income levels. This ensured a broad understanding of family childcare needs, allowed for the identification of macro-level trends, and enhanced the representativeness of the study. In addition, in-depth interviews were conducted with parents of infants and toddlers, as well as with staff from Inclusive and private childcare institutions. These interviews provided insights into parents’ real considerations and demands in choosing childcare services, the operational challenges faced by Inclusive childcare institutions (e.g., facilities, staffing, and funding), and the difficulties encountered by private institutions in market competition and policy adaptation. They also captured the responses and perceptions of various stakeholders under special circumstances such as policy changes and seasonal fluctuations.

### 3.2 Participants

Participants in this study were selected according to clear inclusion and exclusion criteria. The family sample was defined as permanent residents of Guangxi with children aged 0–3 years who were able to complete the survey or interview. The institutional sample consisted of registered Inclusive and private childcare institutions in Guangxi that had been in operation for at least 6 months and were willing to participate. Exclusion criteria included non-residents or temporary residents of Guangxi, institutions with serious legal violations or those that had ceased operation or were under rectification, and individuals unable to communicate effectively due to language or cognitive impairments.

### 3.3 Techniques and tools

A questionnaire titled “Inclusive Childcare Services for Infants and Toddlers Aged 0–3 in Guangxi” was developed, covering dimensions such as basic family information (income, education, ethnicity), childcare demand and preferences, policy awareness, and economic burden. In addition, a semi-structured interview outline was designed for different respondent groups: the parent section addressed factors influencing childcare decision-making and policy experiences, while the organizational section explored operating costs, policy support, and bottlenecks in service provision.

### 3.4 Data collection and analysis

Prior to data collection, participants were informed of the study’s purpose, content, potential risks, and privacy protection measures. Written informed consent was obtained (with electronic confirmation for the online survey). Questionnaires were distributed through both the online platform (Questionnaire Star) and offline field research, with assistance from trained investigators. In-depth interviews were conducted either face-to-face or via video conferencing. To ensure data quality, invalid questionnaire responses were excluded, and interview data were independently coded and cross-verified by two researchers to ensure accuracy.

## 4 Research results

To gain a comprehensive understanding of the actual functioning of childcare services in Guangxi and the real needs of various stakeholders, this study adopts a mixed-methods approach combining questionnaire surveys and in-depth interviews.

The questionnaire survey focuses on parents of children aged 0–3 years, employing standardized instruments to collect extensive data on childcare practices, service preferences, and information access. This helps to identify prevalent issues and emerging trends in childcare demand. In parallel, in-depth interviews were conducted with key representatives, including policymakers, service providers, and users, to obtain more detailed insights through open-ended discussions and to uncover the underlying pain points in policy implementation. By integrating these two methods, the study not only quantitatively reveals the common challenges faced by families, but also qualitatively explores the institutional and operational difficulties encountered in delivering inclusive childcare services. This provides a more robust empirical basis for improving the childcare service system in Guangxi.

### 4.1 Questionnaire survey

#### 4.1.1 Challenges faced by respondents in the process of child care and parenting

##### 4.1.1.1 Expenditures on child care and parenting for 0–3 year olds

**Figure 1:**
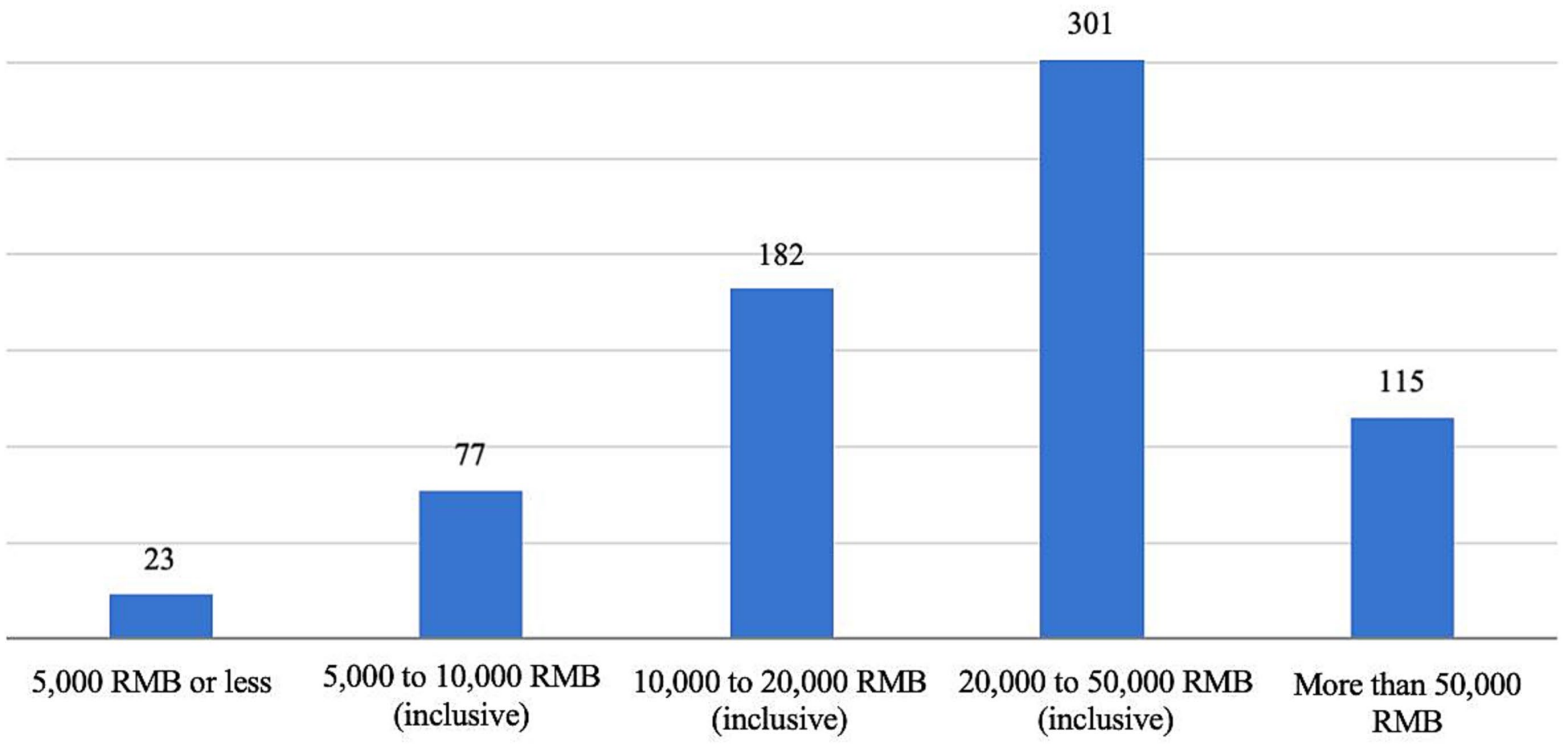
Respondents’ total expenditures on raising and caring for children in the past year.

According to a survey on childcare and parenting, Figure 1 indicates that 301 respondents (43.12%) reported total expenditures on child-rearing and care ranging from 20,000 to 50,000 RMB. Additionally, 182 respondents (26.07%) spent between 10,000 and 20,000 RMB, while 115 respondents (16.48%) reported expenditures exceeding 50,000 RMB. These findings suggest that the costs of raising and caring for children aged 0–3 pose a significant financial burden on families.

**Figure 2:**
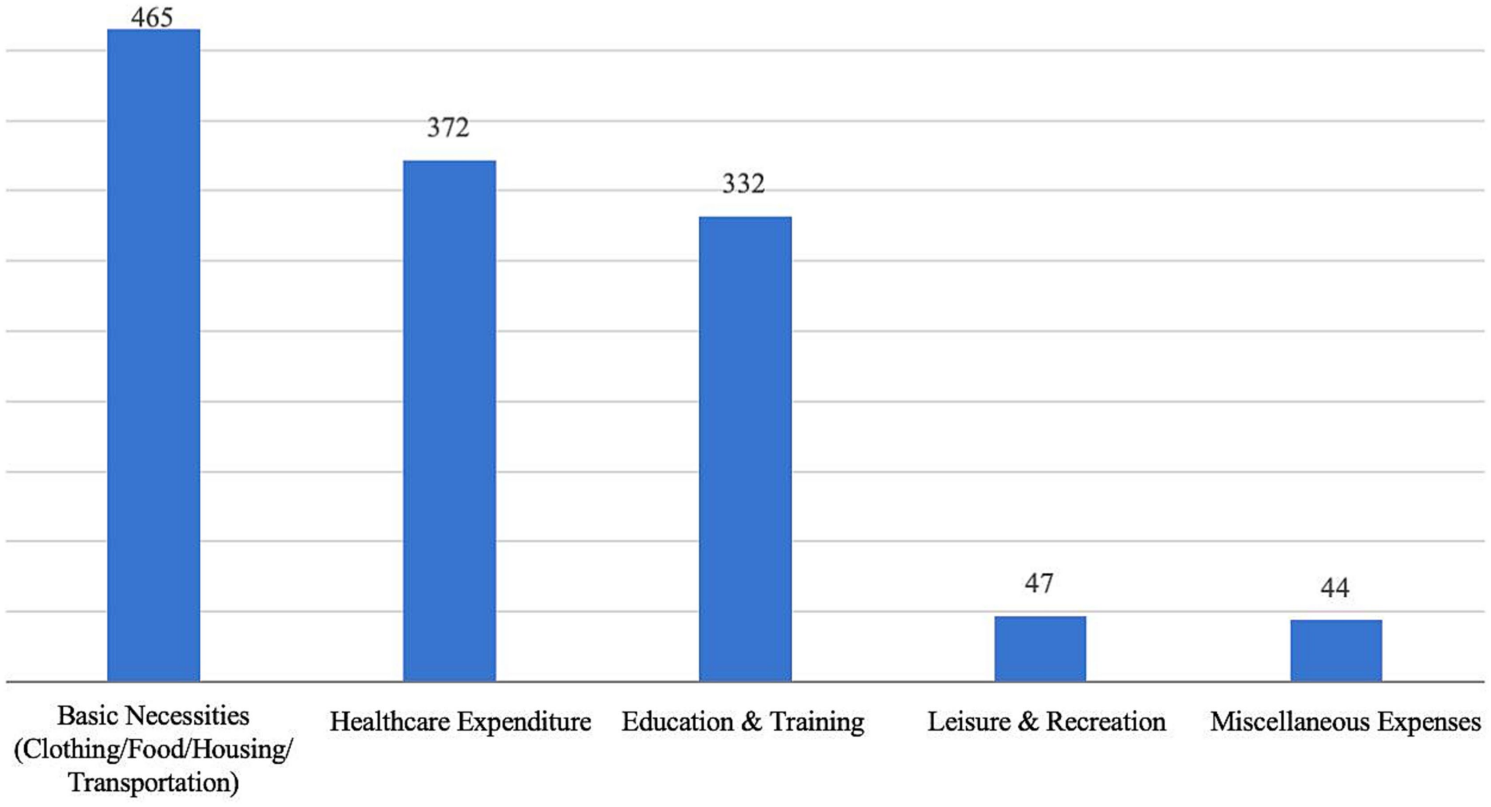
Expenditures on raising and caring for children that account for the largest share of expenditures.

Specifically, 66.62% of respondents identified basic necessities (clothing, food, housing, and transportation) as their biggest expense. Meanwhile, 53.3% cited healthcare expenditures, while 47.56% considered early education and training their primary expense. This suggests that parents face significant financial pressure in raising and caring for young children, and the high cost of childcare may pose a major financial challenge for families. Therefore, expanding access to inclusive childcare services is of great social significance, as it can effectively alleviate the financial burden on families and contribute to sustainable social development (Figure 2).

##### 4.1.1.2 Main difficulties in the re-employment process due to childcare responsibilities

**Figure 3:**
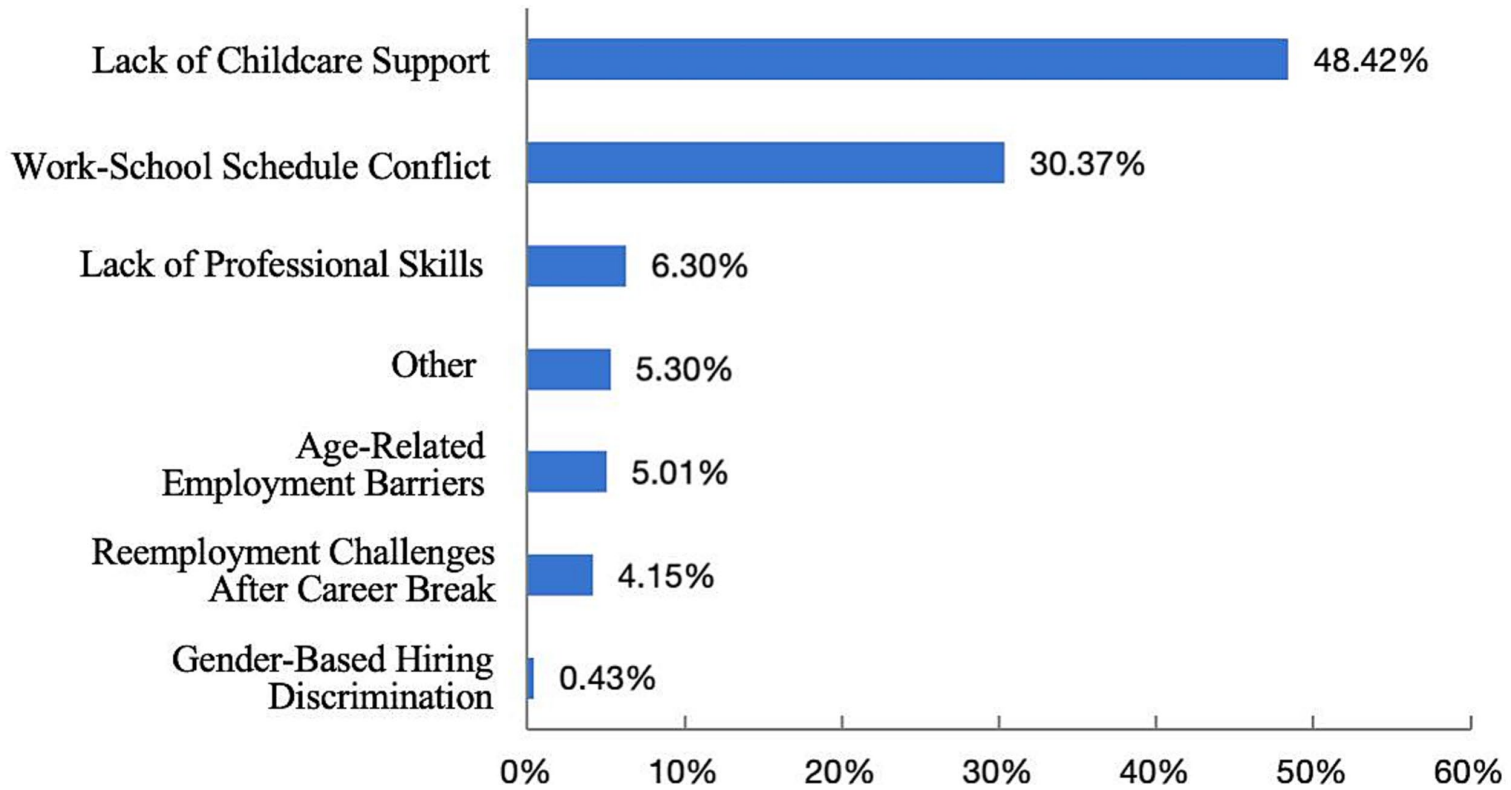
Percentage of respondents with the most significant difficulties in producing/raising/caring for children for re-employment.

Figure 3 shows that 338 respondents (48.42%) identified the lack of childcare support as their primary challenge when re-entering the workforce. In addition, 30.37% of respondents reported conflicts between school drop-off/pick-up times and their work schedules. Other reported challenges include a lack of professional skills (6.3%), age-related employment barriers (5.01%), and reemployment challenges after a career break (4.15%). This indicates that the lack of childcare options is one of the greatest barriers to re-employment, underscoring the severe shortage of inclusive childcare services and leaving numerous families without sufficient support. Meanwhile, young families face significant challenges in balancing work and family responsibilities.

#### 4.1.2 Current situation of childcare and demand for childcare services

##### 4.1.2.1 Ways to get information about childcare services

**Figure 4:**
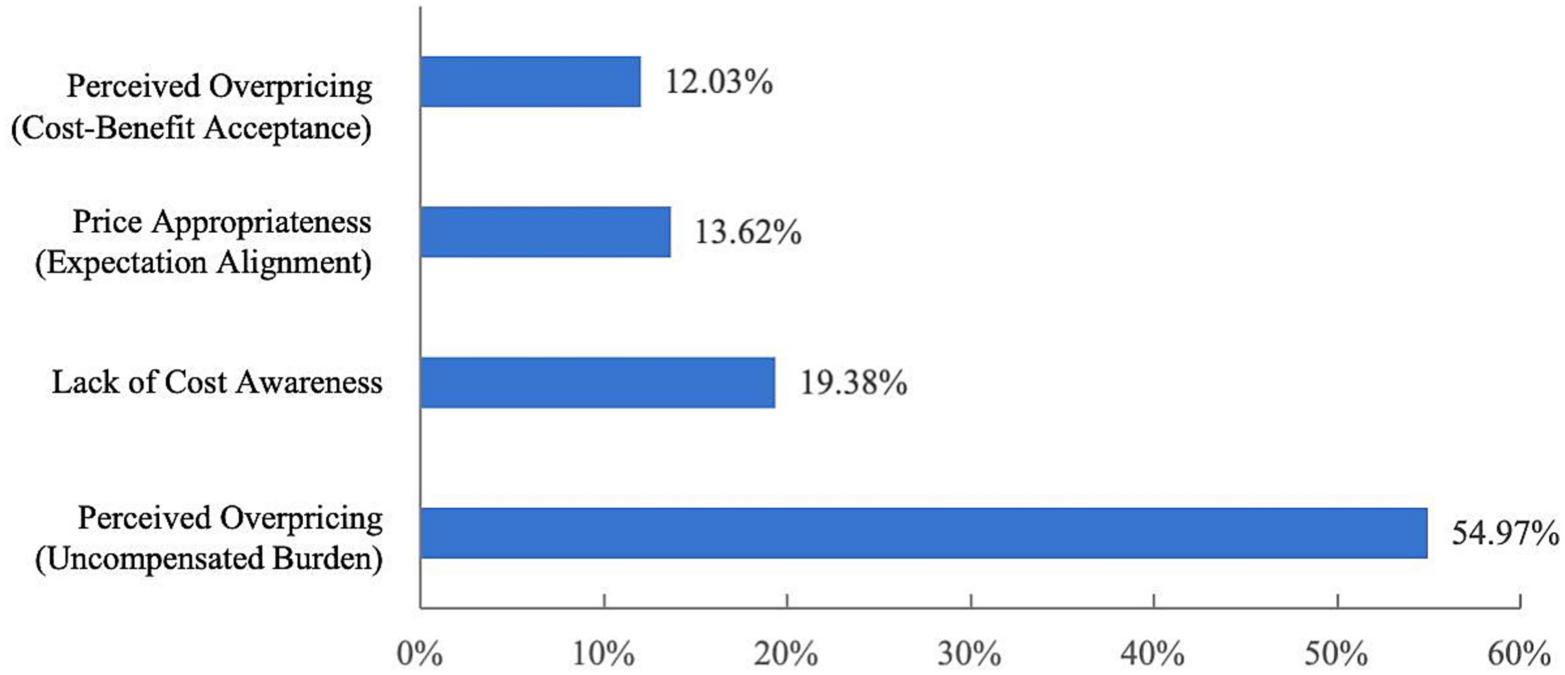
Respondents’ access to information about child care services.

Figure 4 shows that most respondents obtained information about childcare services through various channels, among them 926 respondents reported learning about childcare services through informal networks; 795 respondents obtained information through digital platforms; 443 respondents gained knowledge by browsing the official websites of childcare organizations; and 410 respondents learned about childcare services by participating in community activities. In addition, some respondents still reported having limited knowledge of childcare services. The dissemination of information on infant and toddler childcare services remains limited, highlighting the need to strengthen policy advocacy to enhance public awareness and expand the reach of childcare services.

##### 4.1.2.2 Perception of children entering childcare institutions

**Figure 5:**
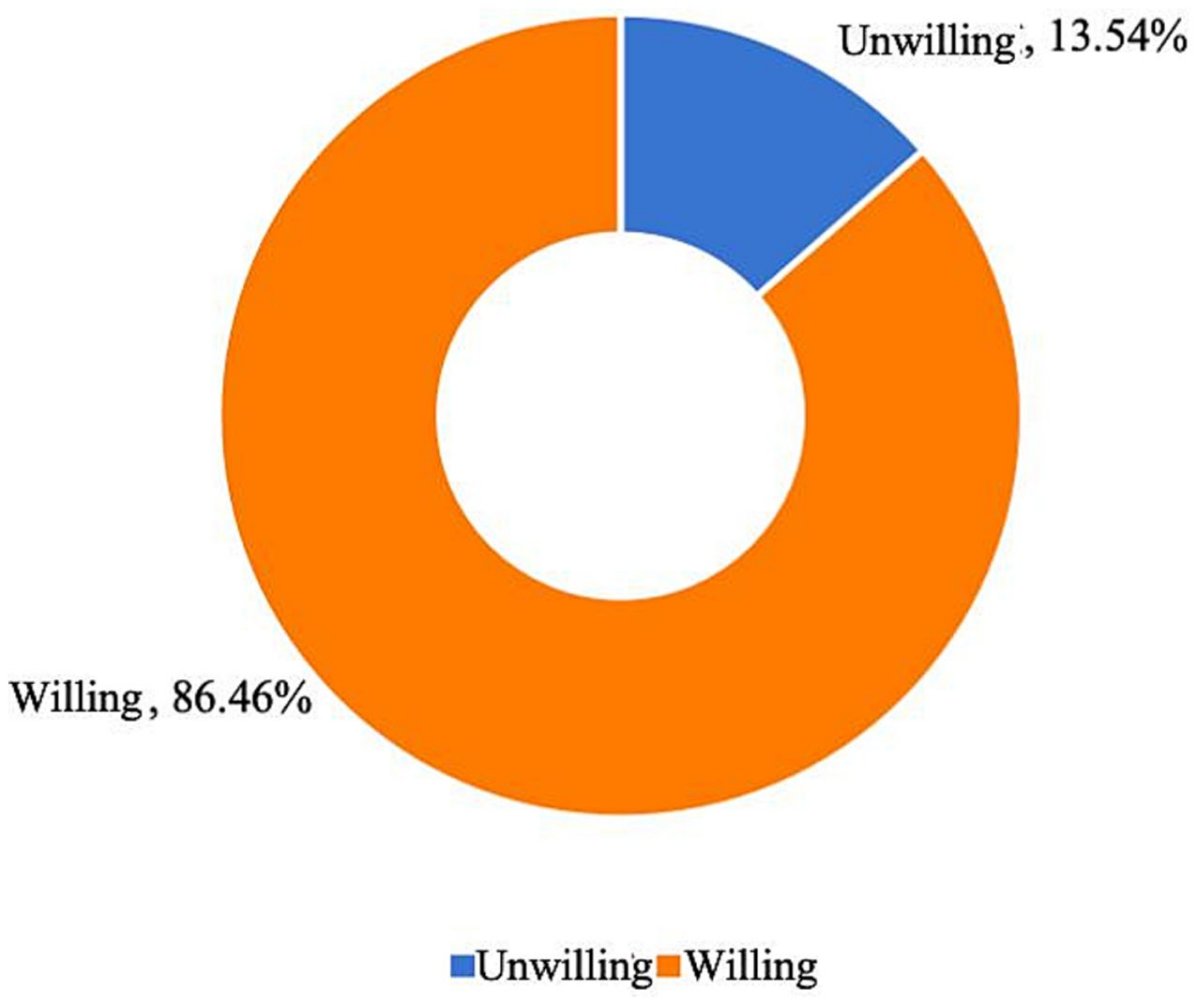
How old do respondents think a child can be admitted to a childcare facility.

Regarding the optimal age for children to enter childcare facilities, Figure 5 indicates that 41.57% of respondents believe children should start at 25–36 months (2–3 years); 22.05% consider 19–24 months appropriate; and 22.77% believe children should begin between 12 and 18 months. A smaller proportion of respondents believe children should enter childcare before 12 months, with 3.67% supporting enrollment before 6 months and 9.94% favoring enrollment between 6 and 12 months. Overall, most parents believe that enrolling children at an older age is safer. This result suggests that parents have concerns about the caregiving capacity of childcare facilities, particularly in terms of safety and reliability.

##### 4.1.2.3 Perception of fees charged by childcare organizations

**Figure 6:**
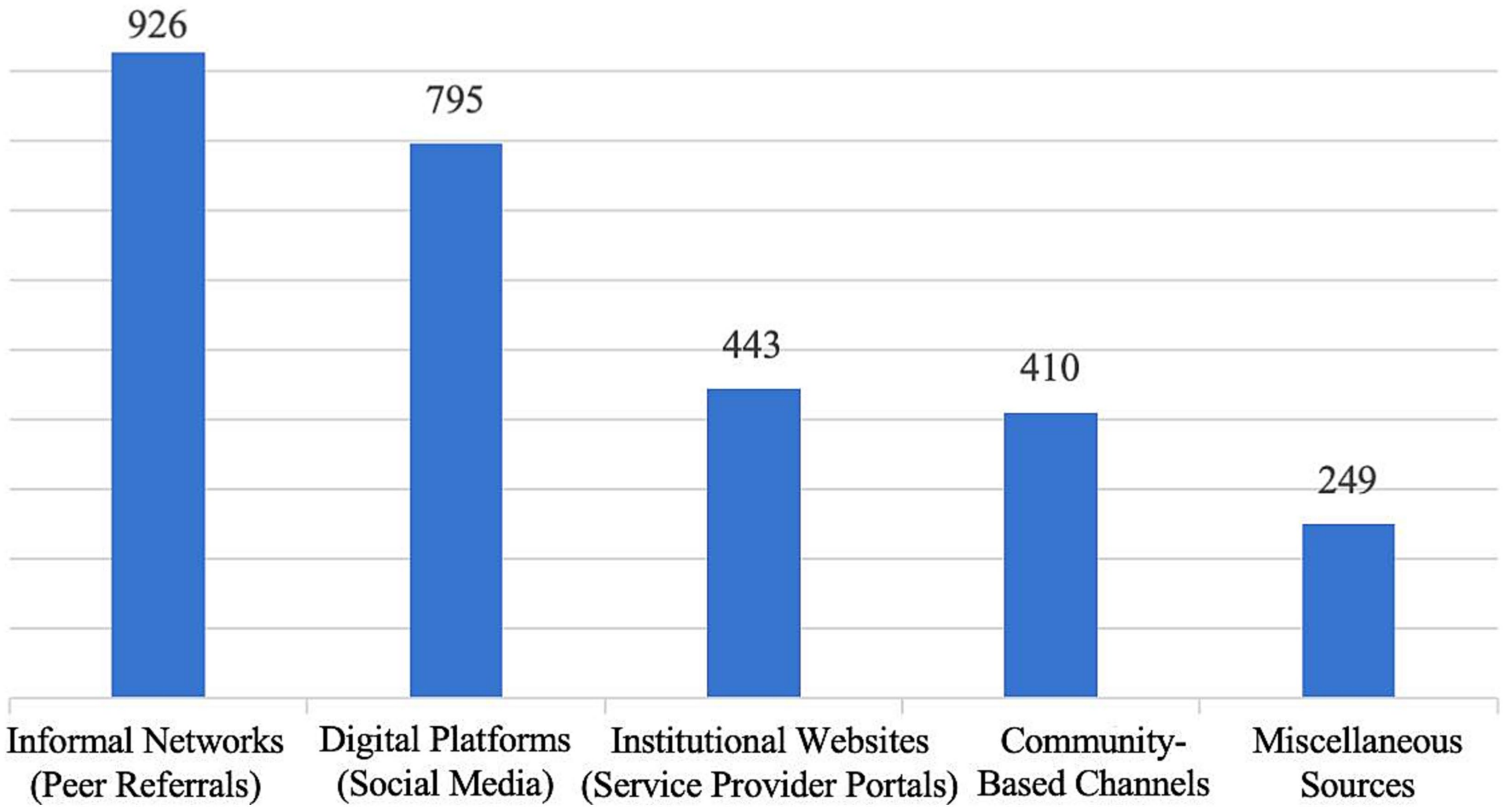
Respondents’ awareness of fees charged by childcare providers.

Figure 6 indicates that more than half of respondents (763 individuals, accounting for 54.97%) believe that childcare fees are not aligned with local economic conditions and are considered relatively high. In addition, 269 respondents (19.38%) reported being unaware of the fee structure, while 167 respondents (12.03%) believed that childcare providers charge high fees, which, despite being misaligned with local economic conditions, are still deemed acceptable. These figures suggest that many families have limited affordability, indicating that childcare pricing may not fully consider the financial realities of different households. The expansion of inclusive childcare system is urgently needed.

##### 4.1.2.4 Main reasons for not sending children to child care institutions

**Figure 7:**
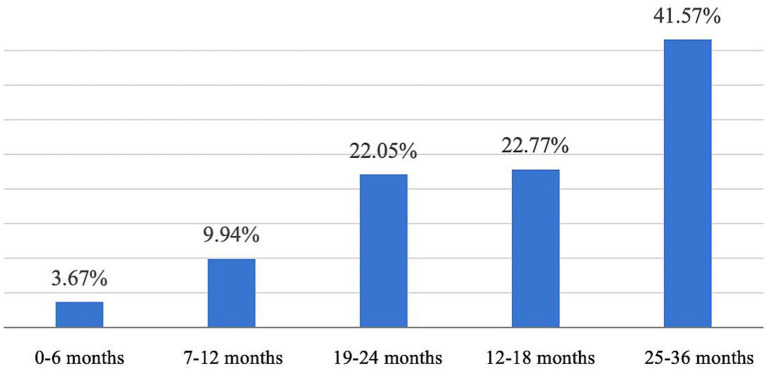
Whether respondents have ever sent their children to a childcare facility.

**Figure 8:**
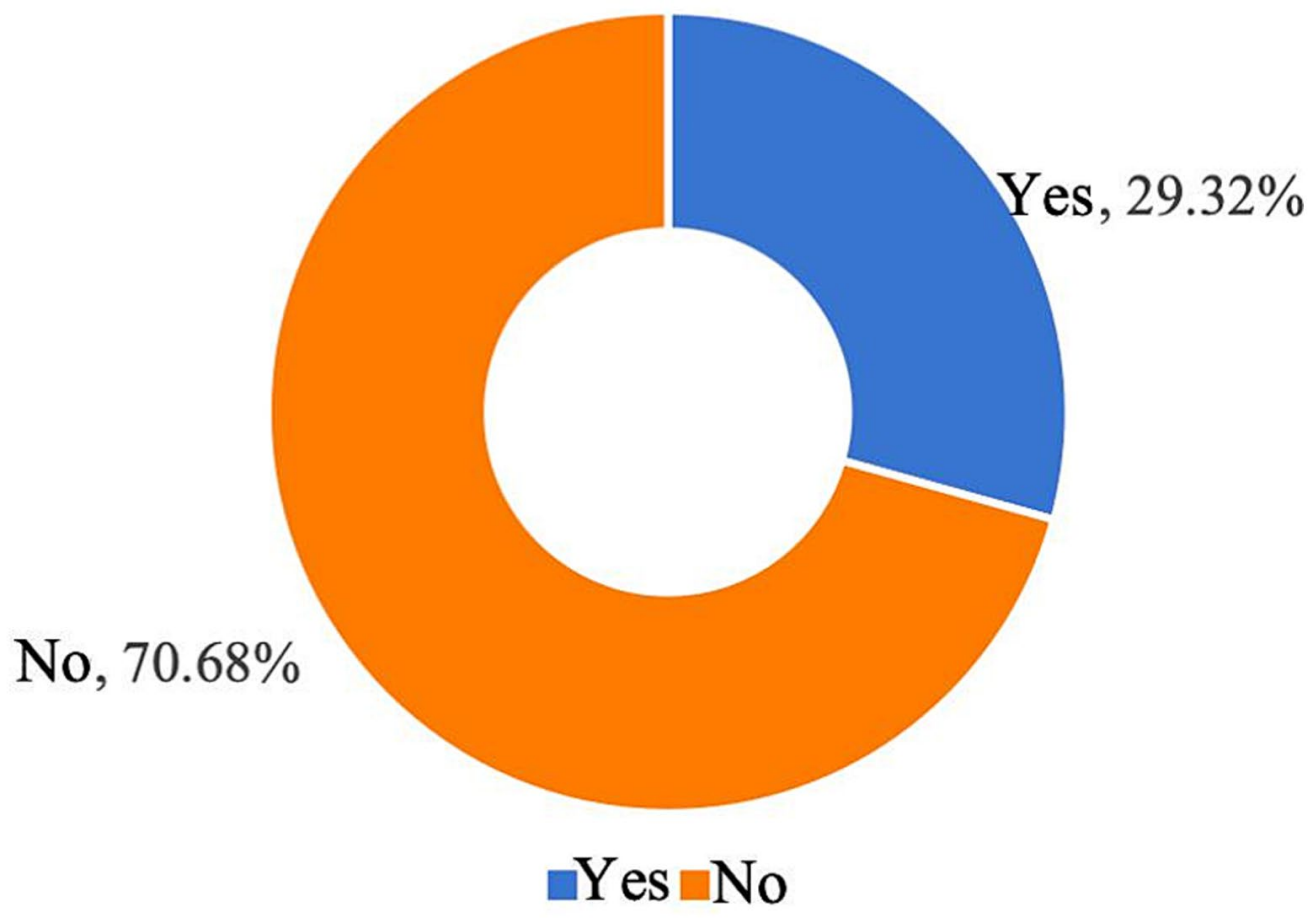
Respondents’ reasons for not sending their children to childcare facilities.

Figure 7 indicates that 981 respondents (70.68%) have not yet enrolled their children in childcare institutions. The main reasons include the high cost of childcare, which many families find unaffordable; the young age of their children, making parents reluctant to separate from them; and concerns about the insufficient professionalism of childcare staff, leading to a lack of trust in childcare institutions (Figure 8). These factors indicate that the local childcare market is characterized by inconsistent service quality and high costs, which impose a significant financial burden on families.

##### 4.1.2.5 Perception and choice of inclusive childcare institutions

**Figure 9:**
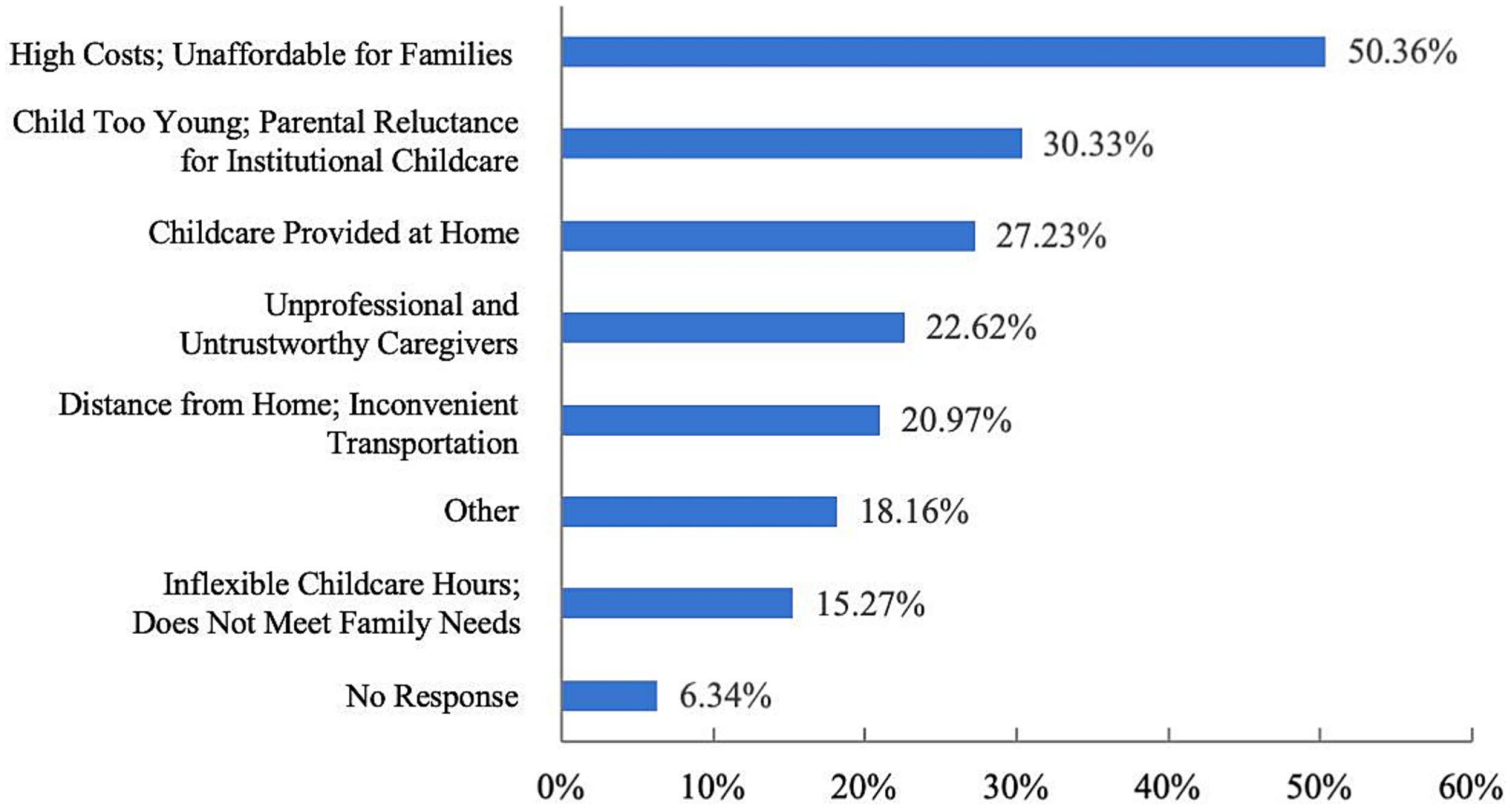
Respondents’ willingness to send their children to a childcare facility.

**Figure 10:**
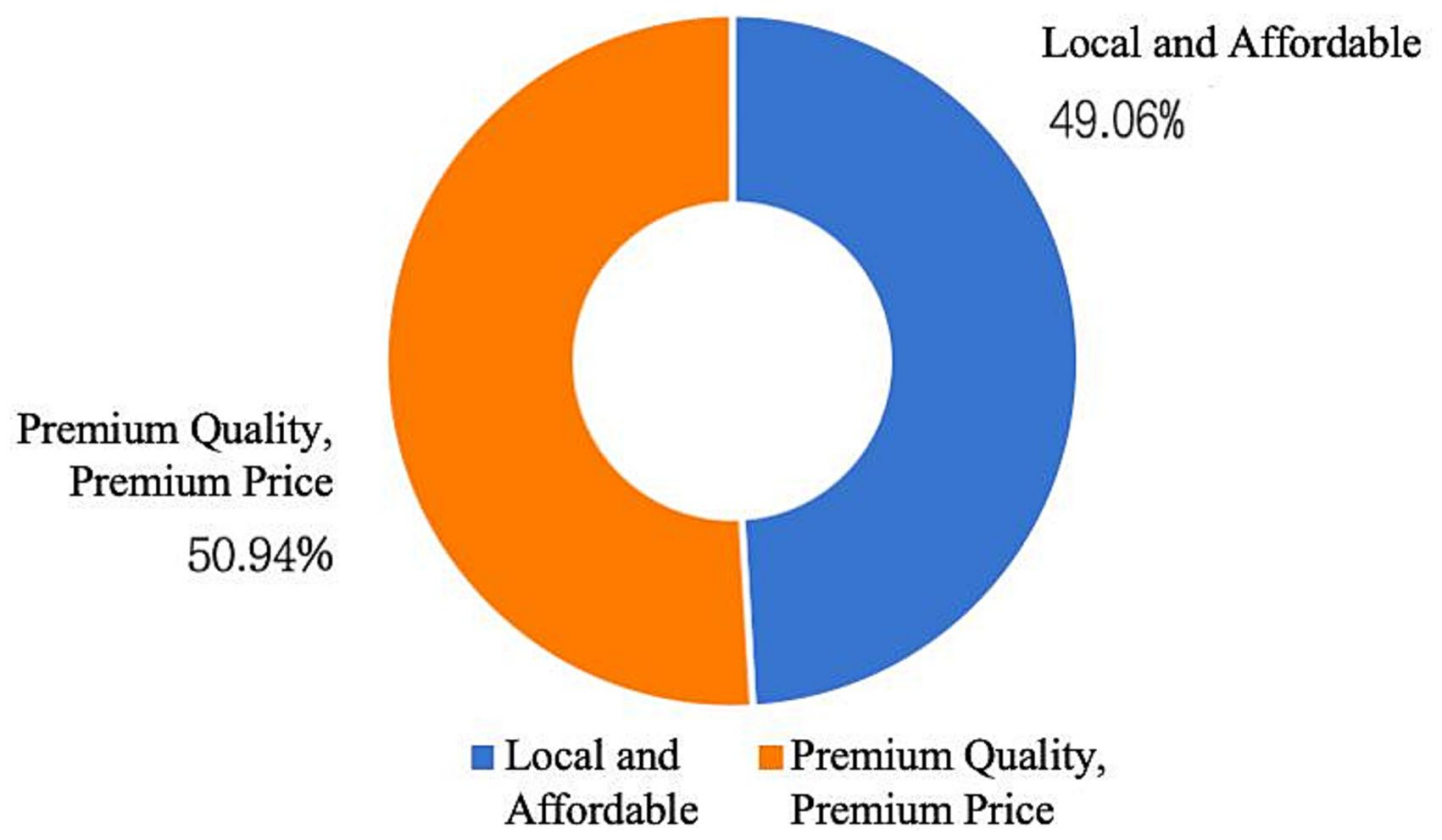
Respondents’ attitudes on whether they would consider having an additional child.

Figure 9 indicates that the majority of respondents (1,200, or 86.46%) would be highly willing to enroll their children under the age of three in childcare institutions if inclusive childcare services were established. In addition, 31.41% of respondents stated that they would consider having another child if inclusive childcare services were available, while 30.26% said they would consider having more children. These findings suggest that the development of inclusive childcare services is closely linked to fertility intentions (Figure 10).

**Figure 11:**
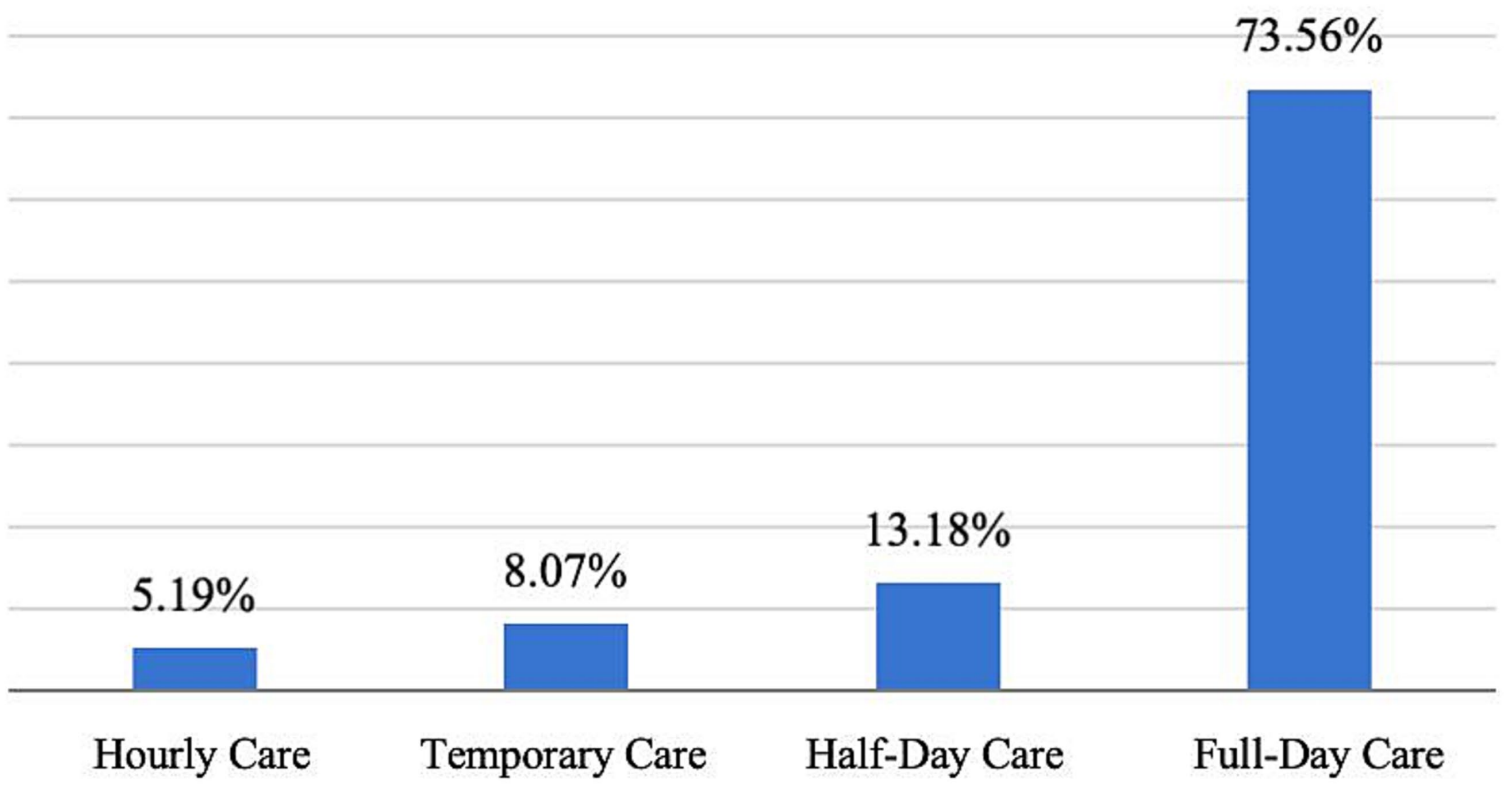
Type of trustee organization preferred by respondents.

**Figure 12:**
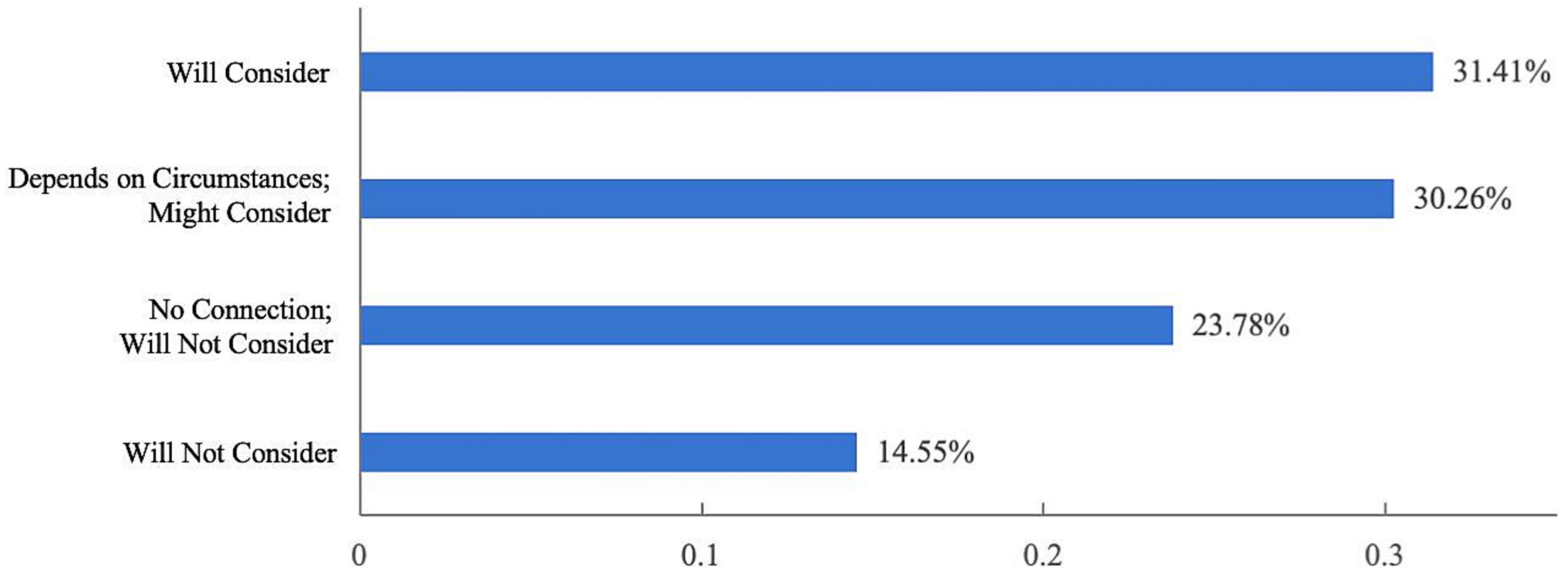
Services respondents want from child care providers.

Figure 11 indicates that 50.94% of respondents (707 in total) preferred premium quality and premium price inclusive childcare institutions, while 49.06% prioritized proximity when choosing a childcare institution. Respondents generally expected childcare institutions, in addition to providing safe childcare services, to expand their services to include a broader range of services such as catering, education, healthcare, and entertainment (Figure 12). This trend reflects the increasing demand for childcare services as society continues to develop.

**Figure 13:**
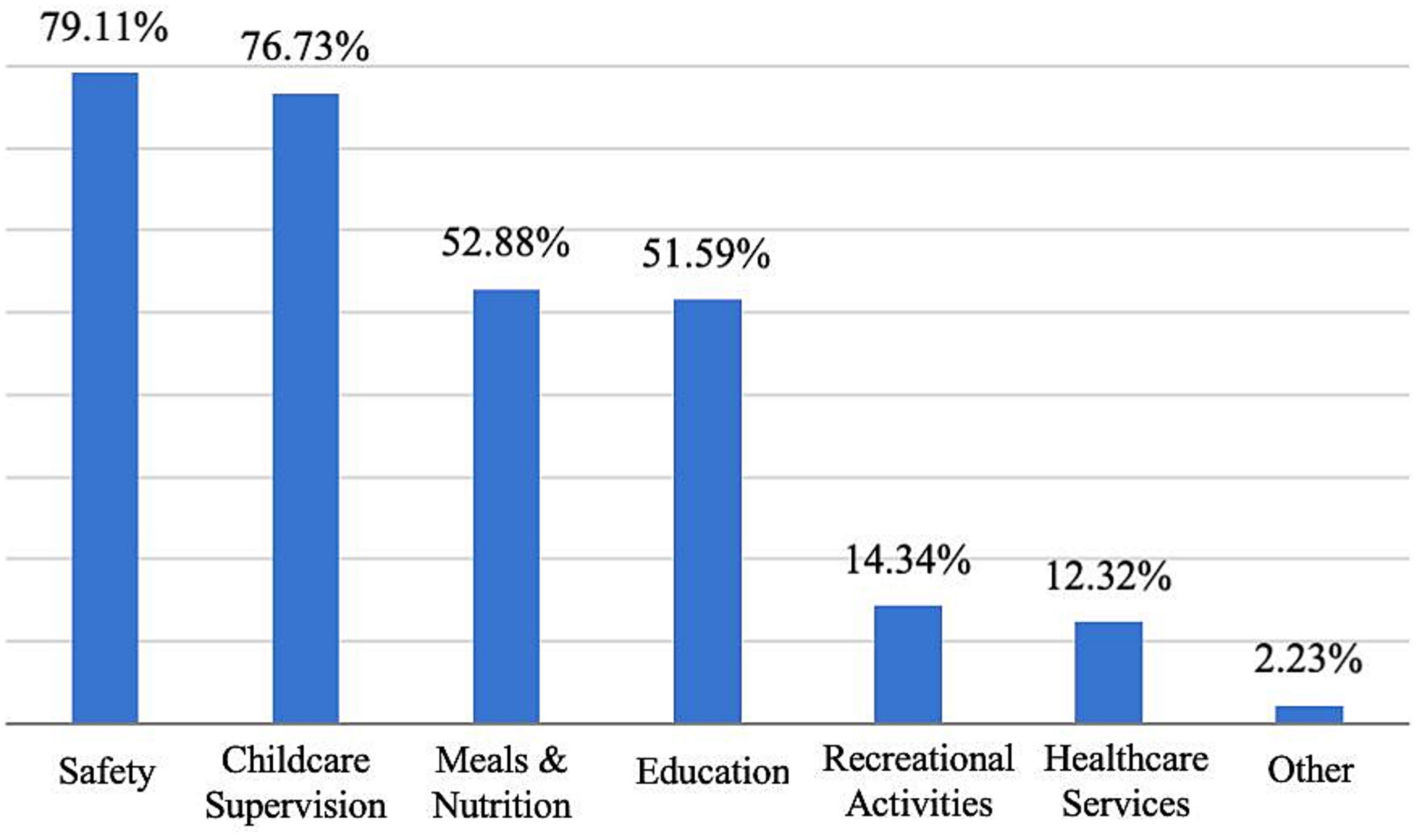
Form of childcare desired by respondents.

Figure 13 indicates that parents demonstrate a strong preference for specific types of childcare services. Full-day childcare is the most popular choice, with 73.56% of respondents choosing this option, as it ensures a structured, standardized, and professional daily care routine for their children. Additionally, 13.18% of respondents preferred half-day care, 8.07% opted for temporary care, and 5.19% selected hourly care. These findings highlight that parents have diverse childcare needs and indicate a demand for more flexible options to better meet the varying needs of families.

**Figure 14:**
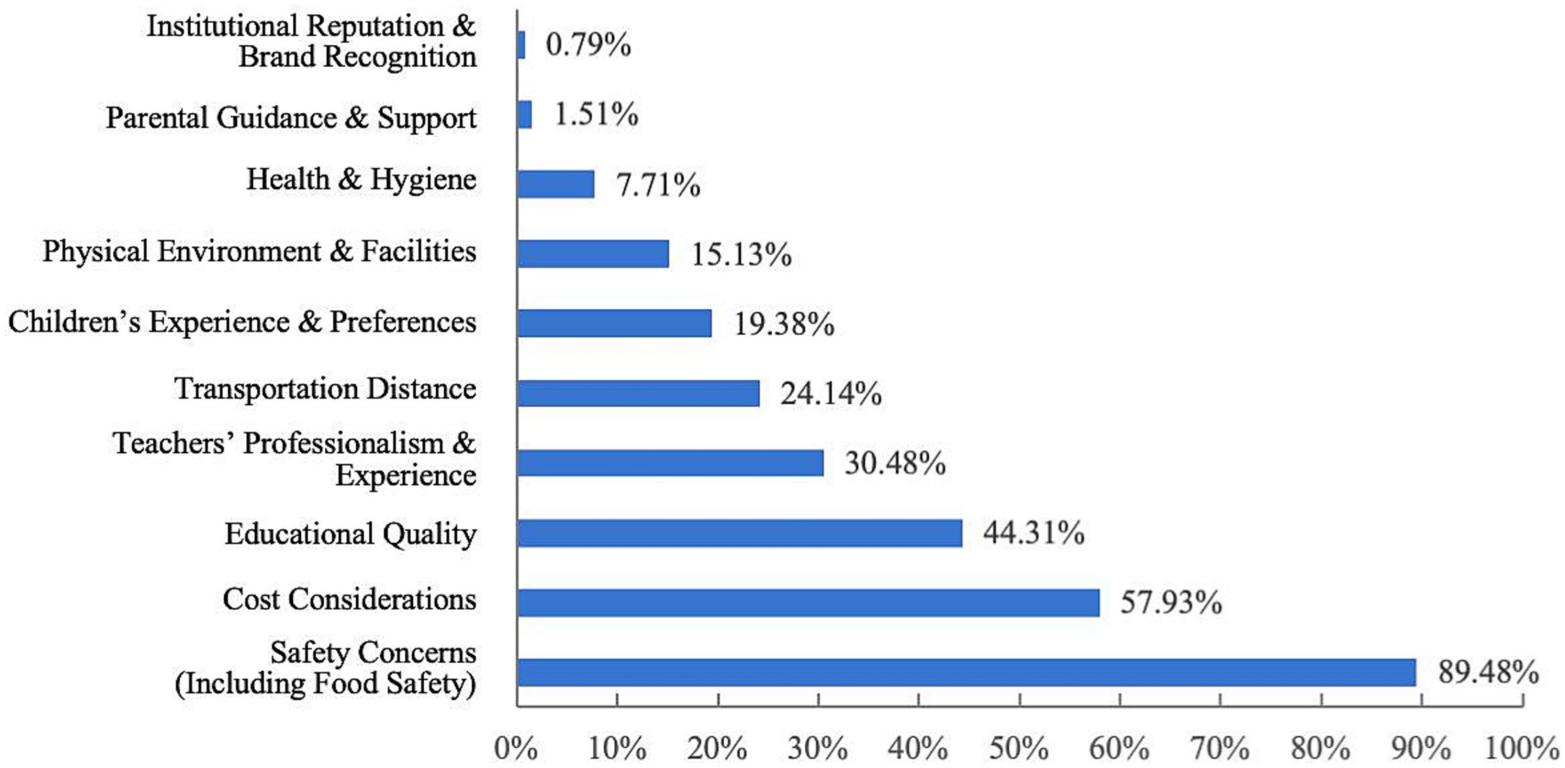
Issues valued by respondents.

The most important factor for respondents is child safety, with food safety being a top concern, followed by cost, education quality, the professionalism and experience of the teaching staff, and transport accessibility. This suggests that parents not only prioritize child safety when choosing a childcare institution but also have diverse requirements for other aspects (Figure 14).

### 4.2 In-depth interviews

#### 4.2.1 Interview participants

This study conducted interviews with ten participants, including parents of infants aged 0–3 years, managers of public (affordable) childcare institutions, managers of private childcare institutions, officials from the childcare-related division of the Health and Wellness Bureau, and representatives from social organizations. Details of the interview participants are shown in Table 1.

**Table 1:** Interview participant information.

No.	Description	Participant code
1	Parent of a child aged 0–3	A01
2	Parent of a child aged 0–3	A02
3	Director of an inclusive childcare institution	B01
4	Director of an inclusive childcare institution	B02
5	Director of a private childcare institution	C01
6	Director of a private childcare institution	C02
7	Staff member, Health and Wellness Bureau (childcare division)	D01
8	Staff member, Health and Wellness Bureau (childcare division)	D02
9	Representative of a social organization	E01
10	Representative of a social organization	E01

#### 4.2.2 Analysis of interview results

##### 4.2.2.1 Parental perspective: genuine demand for childcare services and the dilemma of choice

Interviews with two parents of infants aged 0–3 (A01, A02) and a childcare support worker from a social organization (E01) revealed a strong and genuine demand for childcare services among families in Guangxi. However, in practice, families face the dilemma of “wanting childcare but struggling to access it,” primarily reflected in three areas: high costs, lack of trust, and a mismatch in service provision.

Firstly, the financial burden has become a significant barrier for families seeking childcare. Although subsidized childcare is intended to be affordable, it still represents a considerable expense for most dual-income families. “We came to Guangxi from another province for work. Our two-and-a-half-year-old attends a nearby childcare center. Tuition is 2,600 yuan, and with meals and activities, it totals over 3,300 yuan monthly. Our income is not high—this amount is almost equivalent to my monthly salary.” (A02) The costs are even higher for families choosing private providers. “The private center near our neighborhood starts at 3,600 yuan. There are lots of programs, but it’s simply unaffordable for an ordinary family in the long run.” (A01) This suggests that although parents widely acknowledge the importance of early childcare, financial capacity largely determines whether they can enroll their children.

Secondly, a widespread lack of trust in childcare services persists among parents. Many parents expressed that their reluctance to use childcare services is primarily driven by safety concerns. “Our child is just over 1 year old and cannot talk. If something happens, we would not know. Every day there are news reports of childcare incidents—it’s hard to feel safe sending them there.” (A02) Parents tend to adopt a wait-and-see attitude, especially toward newly established or non-public centers, even when they are nearby and affordable. Moreover, staff quality, responsibility, and professional competence are key factors considered by parents. “It’s difficult to find a truly responsible teacher who genuinely cares for children. Many are temporary and leave within a few months.” (A01) This reflects that current childcare institutions struggle to meet parents’ dual expectations of safety and quality, particularly in staff stability and professionalism.

Thirdly, parents express clear preferences regarding childcare formats, yet actual provision often fails to match their needs. Interviews indicate that over 70% of parents prefer full-day childcare services to accommodate their work schedules. “We leave at 8 a.m. and finish work at 6 p.m. If the center only runs half a day, we just cannot pick up our child on time.” (A01) Some parents have explored flexible options, such as hourly or temporary care, but found them limited in availability, inconsistent in quality, and relatively expensive—adding to the uncertainty of childcare.

##### 4.2.2.2 Institutional perspective: operational challenges and supply bottlenecks in inclusive childcare

Interviews with managers from inclusive (B01, B02) and private (C01, C02) childcare institutions reveal that providers in Guangxi face significant operational pressures, primarily centered on two issues: (1) staffing shortages and high turnover, and (2) weak parental trust coupled with enrolment challenges.

First, structural weaknesses and imbalances in the childcare workforce have become a major constraint on service quality. Inclusive childcare centers often struggle to attract and retain staff, particularly in frontline caregiving roles where turnover is especially high. As B02 noted, “We have 20 children per class, and the standard ratio is two teachers and one carer. But often, we only have one lead teacher and one carer, and sometimes they have to take turns covering shifts.” While some institutions prioritize hiring early childhood education graduates with formal certifications, most applicants lack relevant experience or even basic infant care skills. According to B01, “Childcare jobs are low-paid and lack social recognition, so many staff quit within a few months. On average, we replace workers two to three times a year.” Private providers also find it difficult to attract qualified staff in a competitive labor market. As C02 explained, “Experienced teachers would rather work in private kindergartens or better-paying maternity care centers.”

Second, the lack of parental trust poses a dual challenge of attracting and retaining families. Despite the affordability of inclusive childcare services, parents often equate lower fees with inferior quality, leading to under-enrolment even in well-resourced centers. As B02 observed, “Some parents would rather pay more for private early education centers than trust our newly established facility.” Private providers similarly note that, in the absence of brand recognition or strong word-of-mouth, promotional efforts yield little return. As C02 remarked, “Advertising does not help much when parents do not already trust or know your brand.”

##### 4.2.2.3 Policy perspective: governance challenges and policy pathways for advancing inclusive childcare

In interviews, two staff members from the Guangxi Health and Wellness Bureau’s childcare division (D01, D02) and a representative from a social organization (E02) provided in-depth insights into the current status and governance bottlenecks in the implementation of inclusive childcare policies. They agreed that the system is at a stage where the policy direction is clear but practical implementation remains fraught with difficulties. Three core challenges were identified: (1) fragmented governance, (2) limited grassroots implementation capacity, and (3) the absence of an integrated information platform.

First, a lack of policy coordination has resulted in fragmented governance. Childcare institutions must interact with multiple agencies—ranging from health and education to human resources and market regulation—at stages including site approval, staff registration, hygiene standards, fire safety, and food licensing. As D01 explained, “Although we have issued some guiding opinions at the municipal level, much of the work is still left to subdistrict and community-level authorities. In practice, overlapping responsibilities and poor information flow often leave childcare institutions uncertain about which department is actually in charge.” This multi-agency governance model, combined with inconsistent standards, hampers effective implementation, increases compliance costs, and disproportionately burdens small and newly established private providers.

Second, weak capacity at the grassroots level has emerged as a critical bottleneck in the final stages of implementation. Although national policy encourages embedded childcare services at the community level, many subdistricts and residents’ committees lack professional personnel, adequate space, or financial resources to support such services. As E02 noted, “Our community would genuinely like to establish a childcare center, but we lack appropriate space and staff familiar with operational procedures, so the initiative has been shelved.” Such instances of passive inertia are particularly common in urban fringe areas and rental-based communities, exacerbating the supply–demand mismatch.

Third, the lack of a unified information platform and service guidance mechanisms limits parents’ awareness and choice. In Nanning, parents still primarily rely on word-of-mouth, social media, and official government notices, which offer limited reach and fragmented information. As D01 stated, “We are working to consolidate all registered childcare institution data citywide and aim to establish a one-stop inquiry platform that includes institution types, fee schedules, government subsidies, and user satisfaction feedback.” Such a platform would not only enhance parents’ decision-making efficiency but also improve institutional self-regulation and facilitate more precise government oversight.

## 5 Discussion

This study focuses on Inclusive childcare services for children aged 0–3 in Guangxi. Using a combination of questionnaire surveys and in-depth interviews, it systematically examines the current supply–demand situation and major problems of childcare services, offering both practical relevance and research depth. The contributions of this study can be summarized in three aspects. First, theoretical contribution: This study systematically applies Amartya Sen’s capability approach to the construction of Inclusive childcare services, moving beyond the traditional emphasis on “resource supply” in childcare research. It offers a novel analytical lens and theoretical foundation for accurately identifying the real needs of different groups in childcare and broadens the analytical perspectives of policy evaluation. Second, policy implications: The study reveals the real challenges of inclusive childcare in Guangxi and proposes optimisation strategies grounded in the capability approach, providing valuable reference for government policy design. Third, practical value: It provides useful reference experiences for promoting inclusive childcare in other less developed regions.

### 5.1 Limitations

This study has three main limitations. First, the survey sample was limited to Guangxi and did not extend to regions with different levels of economic development and marked urban–rural disparities. Second, data collection relied primarily on questionnaires and in-depth interviews, which were useful for identifying key issues but lacked systematic analysis of childcare institutions’ operational data and policy implementation processes, potentially limiting the analytical depth of the study. Third, the analysis of informal family care (e.g., grandparent care) remains limited, warranting further exploration in future research.

## 6 Conclusion

### 6.1 Policy recommendations

Guided by the Capability Approach, this study explored the development of Inclusive childcare services for infants and toddlers aged 0–3 in Guangxi. The findings reveal that the region’s inclusive childcare policies have, to some extent, expanded families’ childcare choices and laid a preliminary foundation for achieving the goal of “ensuring childcare for all.” However, as evidenced by both the questionnaire and interview results, the policy implementation process continues to face a number of pressing challenges. These unresolved issues have hindered the full realization of the policy’s intended function-namely, enhancing families’ feasible capabilities for childcare. As a result, there remains a notable gap between the aspirational goal of “small childcare enabling great happiness” and the lived reality of many families. In light of these findings, the following policy recommendations are proposed:

#### 6.1.1 Strengthening policy empowerment: establishing a capability-oriented support mechanism for inclusive childcare

##### 6.1.1.1 Increasing fiscal investment and targeted subsidies

First, a “Family Childcare Capability Account” should be established to provide need-based childcare subsidy vouchers to low-income families-particularly those in rural or multi-ethnic communities-so as to alleviate financial constraints on their childcare choices. The subsidy amounts should be dynamically adjusted according to urban–rural disparities (e.g., offering higher subsidy ratios for rural families) to ensure that the policy effectively reaches those most in need. Second, operational subsidies for childcare institutions should be optimized. Inclusive childcare providers-especially those located in communities and rural areas-should receive benefits such as rent exemptions, discounts on utilities, and tax reductions, thereby enabling them to reduce fees and increase accessibility.

##### 6.1.1.2 Improving tiered pricing and dynamic supervision to ensure accessibility and service quality

On one hand, a tiered pricing framework should be developed for inclusive childcare services, with maximum allowable fees differentiated for urban, county, and rural areas based on regional economic conditions. This would help prevent excessive market-driven profit-seeking. On the other hand, service quality supervision should be enhanced. Regular public disclosure of safety, hygiene, and quality assessments should be implemented to ensure that service provision remains standardized and trustworthy.

#### 6.1.2 Optimizing the supply structure: building a diversified and tiered inclusive childcare service system

##### 6.1.2.1 Innovation of the “universal+” service model for differentiated and flexible family needs

In response to the questionnaire findings highlighting families’ core demands for “affordable fees” and “childcare aligned with working hours,” and to interview insights revealing supply–demand mismatches such as insufficient full-time coverage and limited flexible childcare, the service model was restructured in two main ways. On the one hand, a tiered supply system should be developed to meet diverse family needs. In communities and rural areas, childcare centers can provide full-day and half-day services with a focus on basic safety and early learning. In urban and county-level cities, an integrated “childcare plus early education” model should be promoted, incorporating culturally relevant elements such as Zhuang language literacy and ethnic cultural experiences. These models should maintain quality standards while ensuring affordable pricing. On the other hand, flexible service formats should be expanded to match the varying needs of families. Hourly care and temporary care options should be more widely available to increase accessibility, convenience, and responsiveness to dynamic caregiving demands.

##### 6.1.2.2 Strengthening urban–rural resource coordination to bridge service gaps

On the one hand, the construction of “community-embedded” childcare networks has been promoted to improve service accessibility. In response to the situation of “few and distant childcare facilities” in old urban communities and the absence of “dedicated childcare facilities” in rural market towns, unused public spaces such as community activity centers and rural cultural stations were repurposed into inclusive childcare centers. This initiative prioritized “service gap areas” including old urban neighborhoods and rural market towns, enabling families to access childcare nearby and addressing the pain point of “distant facilities and inconvenient pick-up and drop-off.” At the same time, reusing public facilities reduced construction costs and ensured that service fees remained affordable. On the other hand, an urban–rural childcare support mechanism has been established to narrow the quality gap in service provision. To address shortcomings in rural childcare institutions caused by “weak teaching staff and unstandardized management,” and to respond to rural families’ demand for more professional childcare services, high-quality urban childcare institutions were encouraged to partner with rural ones. Through measures such as regular teacher training, standardized curriculum sharing, and the transfer of management expertise, rural institutions were systematically upgraded in caregiving capacity, teaching standards, and management efficiency. This gradually narrowed the gap in service quality between urban and rural areas, enabling rural families to access “safe, professional, and inclusive” childcare services and alleviating the long-standing problem of “urban–rural supply–demand imbalance.”

#### 6.1.3 Enhancing feasibility: empowering families and children for multidimensional development

##### 6.1.3.1 Enhancing families’ economic capability

Policies should support families by reducing the financial burden of childcare. Childcare expenses could be made tax-deductible under individual income tax schemes, or childcare allowances could be introduced to directly offset costs. In addition, enterprises that establish workplace childcare centers or offer childcare subsidies could be incentivized through tax breaks or preferential policy evaluations, encouraging shared responsibility for caregiving between employers and families.

##### 6.1.3.2 Enhancing families’ social capability

On the one hand, policy outreach must be strengthened. Channels such as social media, neighborhood bulletin boards, parent WeChat groups, and short video platforms should be leveraged to effectively disseminate information and increase awareness among target families. On the other hand, the intrinsic development of children should be promoted. Early education programs developed by Guangxi universities can integrate cultural elements from ethnic groups such as the Zhuang and Yao, including folk rhymes and traditional crafts, to foster both cognitive development and cultural identity in young children.

### 6.2 Future prospects

In the future, research could broaden its scope to include cross-regional comparative studies, thereby exploring the commonalities and differences in the implementation of Inclusive childcare policies across regions and improving the generalisability of the findings. Big data analytics could also be employed to provide a more comprehensive analysis of policy implementation dilemmas by integrating multi-source data, including the operation of childcare institutions and policy processes. Furthermore, the application of the capability approach could be expanded to examine in greater depth how policies affect families’ psychological, temporal, and other dimensions of capability, thereby enriching the theoretical implications of this research. In addition, given the unique characteristics of ethnic regions, future research could focus specifically on the integration of ethnic culture into childcare services, in order to explore locally grounded pathways for childcare development.

## Data Availability

The original contributions presented in the study are included in the article/Supplementary material, further inquiries can be directed to the corresponding authors.
