# Supplementary material for: How can “small childcare” support “great happiness”? A study on inclusive childcare services for infants and toddlers aged 0–3 in Guangxi through the lens of the capability approach
Source: Front Public Health. 2025 Oct 14;13:1664735. doi: 10.3389/fpubh.2025.1664735 (PMC12558841; doi:10.3389/fpubh.2025.1664735)
Supplement: Supplementary file 1 [file Table_1.DOCX]

Supplementary Material

# Survey on Inclusive Childcare Services for Infants Aged 0-3 in Guangxi from the Perspective of Capability Approach

Part One: Personal Background Information

1.What is your gender?

A. Male  B. Female

2，Date of birth: ______ year ______ month

3.What is your current place of residence?

A. Urban area of a prefecture-level city

B. County-level city

C. County seat

D. Township

4.What is your ethnic group?

A. Han  B. Zhuang  C. Yao  D. Other minority ethnic group: _______

5.What is your marital status?

A. Unmarried  B. Married

6.What is your highest level of education?

A. Senior secondary education (high school/technical/vocational)

B. Associate degree

C. Bachelor's degree

D. Master's degree

E. Doctorate

F. Other: _______

7.What is the nature of your workplace?

A. Government agency/public institution

B. State-owned enterprise

C. Non-state-owned enterprise

D. Other: _______

Part Two: Current Family Childcare Situation

8.Do you have infants aged 0–3 in your household?

A. Yes (continue)  B. No (end the questionnaire)

9.Do you live with the infant(s)?

A. Yes  B. No

10.What is your relationship with the child?

A. Parent  B. (Grand)parent  C. Other: _______

11.Who primarily cares for the child on a daily basis? (Select all that apply)

A. Father  B. Mother  C. (Grand)parents  D. Nanny/domestic worker  E. Other: _______

12.Approximately how much did your household spend on childcare in the past year?

A. RMB 5,000 or less

B. RMB 5,000–10,000

C. RMB 10,000–20,000

D. RMB 20,000–50,000

E. Over RMB 50,000

13.What is the largest childcare-related expenditure in your family?

A. Daily necessities (food, clothing, housing, transport)

B. Medical and health care

C. Education and entertainment

D. Childcare services

E. Other: _______

14.What do you consider to be the main obstacles to re-employment due to childcare? (Select all that apply)

A. Lack of professional skills

B. Age-related employment difficulty

C. Schedule conflict between work and childcare

D. Inconvenient childcare services

E. No one to help with childcare

F. Other: _______

Part Three: Awareness and Demand for Childcare Services

15.How do you typically obtain information about childcare services? (Select all that apply)

A. Recommendations from family and friends

B. Community bulletin boards

C. Government websites or official social media accounts

D. On-site consultations at childcare institutions

E. Other: _______

16.At what age (in months) do you think a child is suitable to attend a childcare institution?

A. 6 months or younger

B. 7–12 months

C. 13–18 months

D. 19–24 months

E. 25–36 months

17.Have you ever sent your child to a childcare institution?

A. Yes (please answer questions 18–19)

B. No (please answer questions 20–21)

18.What are the main reasons you chose a childcare institution? (Select all that apply)

A. To support employment

B. Scientific parenting guidance

C. To reduce family care burden

D. To promote child’s social development

E. No one at home to care for the child

F. Other: _______

19.How satisfied are you with the current childcare services?

A. Very satisfied  B. Satisfied  C. Neutral  D. Dissatisfied  E. Very dissatisfied

20.What are the main reasons you did not choose a childcare institution? (Select all that apply)

A. Fees are too high

B. Inconvenient transportation

C. Lack of trust in service quality

D. Child is too young

E. Inflexible schedule

F. Someone at home can care for the child

G. Other: _______

21.Do you think the fees for childcare services are higher than you expected?

A. Reasonable

B. Higher than expected

C. Higher than expected but acceptable

D. Not sure

22.What are your core expectations for inclusive childcare institutions? (Select all that apply)

A. High quality at reasonable price

B. Proximity and accessibility

23.If affordable and nearby inclusive childcare institutions become widely available, would you be willing to use them?

A. Yes  B. No

24.What type of childcare services do you prefer?

A. Full-day care  B. Half-day care  C. Temporary care  D. Hourly care

25.If there were affordable, nearby inclusive childcare institutions, would you consider (or recommend) having another child?

A. Yes  B. No  C. It does not matter

# Interview Guidelines for Universal Infant Care Services for Children Aged 0–3 in Guangxi from the Perspective of Capability Approach

Dear Respondent,

Hello! We are students from Guangxi University conducting research on the development of infant care services in the Guangxi region. This interview aims to gain a deeper understanding of how current childcare policies are being implemented and to explore the real needs of different sectors of society. Our goal is to provide research-based support and policy recommendations to help optimise and improve the childcare service system in Guangxi.

We sincerely appreciate you taking the time out of your busy schedule to participate in this interview. All information collected will be used solely for academic research and will remain strictly confidential. No personal information will be disclosed without your consent. We look forward to your honest and valuable insights based on your personal experience and observations.

Thank you once again for your support and trust!

1. Interview with Parents of Infants Aged 0-3
2. Does your family currently have a need for childcare services? Have you already selected a childcare institution?
3. What aspects are most important to you when choosing childcare services? (e.g., cost, safety, distance, staff qualifications)
4. What type of childcare arrangement do you prefer? (e.g., full-day care, half-day care, temporary care) Why?
5. What are your main sources of information about childcare institutions? Are you familiar with the inclusive childcare policy?
6. What do you consider to be the biggest challenges or dissatisfactions with current childcare services?
7. Interview with Directors of Inclusive and Private Childcare Institutions
8. Could you briefly describe the basic situation of your institution? (e.g., target group, class size, fee standards)
9. What are the main operational challenges your institution currently faces? (e.g., staffing, enrolment, funding)
10. Where do your teachers and childcare staff mainly come from? What difficulties do you face in recruitment and retention?
11. Have you applied for any government subsidies related to inclusive childcare policies? Was the application process smooth?

5.How would you describe the current level of trust parents have in childcare institutions? What misunderstandings or expectations do they have?

III. Interview with Staff from Childcare-Related Departments of the Health and Wellness Bureau and Representatives from Social Organizations

1.What responsibilities does your department/organization currently undertake in promoting inclusive childcare services?

2.What are the major implementation challenges or bottlenecks faced during policy rollout or support?

3.What gaps still exist in the dissemination of childcare information and parent guidance in Nanning City?

4.Do communities currently have the basic conditions necessary to develop embedded childcare services? (e.g., facilities, human resources)

5.What key measures or recommendations do you believe are essential for the sustainable development of inclusive childcare services?
